# Generation and evaluation of artificial mental health records for Natural Language Processing

**DOI:** 10.1038/s41746-020-0267-x

**Published:** 2020-05-14

**Authors:** Julia Ive, Natalia Viani, Joyce Kam, Lucia Yin, Somain Verma, Stephen Puntis, Rudolf N. Cardinal, Angus Roberts, Robert Stewart, Sumithra Velupillai

**Affiliations:** 1 Department of Computing, Imperial College London, London, SW7 2AZ UK; 2 IoPPN, King’s College London, SE5 8AF London, UK; 3 Department of Psychiatry, University of Oxford, Warneford Hospital, OX3 7JX Oxford, UK; 4 Department of Psychiatry, University of Cambridge, Downing Street, Cambridge, CB2 3EB UK; 5 Cambridge Biomedical Campus, Cambridgeshire and Peterborough NHS Foundation Trust, Box 190, Cambridge, CB2 0QQ UK; 6 South London and Maudsley NHS Foundation Trust, SE5 8AZ London, UK

**Keywords:** Medical research, Scientific community

## Abstract

A serious obstacle to the development of Natural Language Processing (NLP) methods in the clinical domain is the accessibility of textual data. The mental health domain is particularly challenging, partly because clinical documentation relies heavily on free text that is difficult to de-identify completely. This problem could be tackled by using artificial medical data. In this work, we present an approach to generate artificial clinical documents. We apply this approach to discharge summaries from a large mental healthcare provider and discharge summaries from an intensive care unit. We perform an extensive intrinsic evaluation where we (1) apply several measures of text preservation; (2) measure how much the model memorises training data; and (3) estimate clinical validity of the generated text based on a human evaluation task. Furthermore, we perform an extrinsic evaluation by studying the impact of using artificial text in a downstream NLP text classification task. We found that using this artificial data as training data can lead to classification results that are comparable to the original results. Additionally, using only a small amount of information from the original data to condition the generation of the artificial data is successful, which holds promise for reducing the risk of these artificial data retaining rare information from the original data. This is an important finding for our long-term goal of being able to generate artificial clinical data that can be released to the wider research community and accelerate advances in developing computational methods that use healthcare data.

## Introduction

Natural Language Processing (NLP) can potentially improve healthcare by facilitating analysis of unstructured text. A key obstacle to the development of more powerful NLP methods in the clinical domain is data accessibility, mainly due to ethical constraints on sharing documents that contain personal information, such as electronic health records (EHRs)^1^. There have been efforts to make de-identified EHR data available for research, but these usually come with strict governance regulations. There are also very few resources specific to mental health available. In the machine learning community, similar problems are typically solved by using artificially generated data, e.g., Bachman, Gulrajani et al.^2,3^ in image processing. Text generation is an active area of NLP research covering tasks, such as dialogue generation, machine translation (MT), summarisation, and story generation.

Generation of medical data destined to help clinicians in their daily work is a commonly addressed issue^4,5^. For instance, Jing et al.^4^ tackle the generation of medical imaging reports (up to 50 words), using a hierarchical recurrent neural network decoder. The decoder generates a sequence of topic representations conditioned on image and image tag information. Each representation then conditions the generation of respective sentences. Another example is the study by Liu^5^, where generative models are used to predict the content of EHR notes conditioned on past patient data. However, the replacement of genuine training data with artificial training data remains understudied.

The attempt closest to ours is the one of Lee^6^. They generate short-length (<20 tokens) chief complaint documents, using diagnosis, patient- and admission-related information as conditions. They employ a fairly simple encoder–decoder (ED) architecture. The clinical validity of the generated text is investigated by using it as test data for NLP models built with real data.

The utility of the generated data for downstream NLP tasks is rarely analysed. Furthermore, few studies investigate to what extent these models retain rare information from the original data—rare information could potentially contain sensitive information. To our knowledge, there have been no attempts to automatically generate full EHR notes for NLP purposes. Here, we focus on the generation also of mental health records (MHRs), an understudied clinical domain, and EHR type. Compared to other clinical domains, MHRs are characterised by a greater extent of complex narrative and rely less on structured coding.

**Fig. 1:**
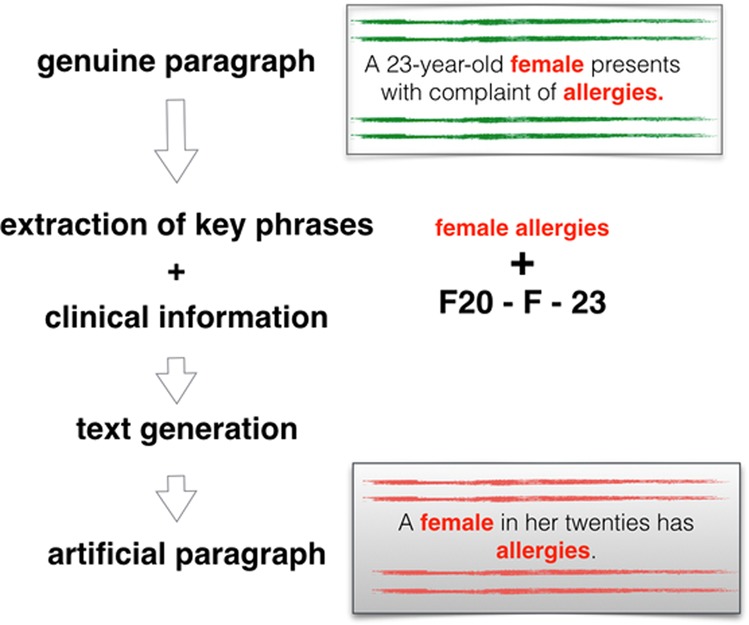
Overview of the text generation procedure. Key phrases are extracted from paragraphs in the original data (genuine paragraph), and combined with clinical information (ICD-10 diagnosis code, gender and age). This is used in our text generation model, producing an artificial paragraph.

In this work, we create artificial medical data using state-of-the-art text generation models. We guide the generation of EHRs with the help of key phrases. These key phrases are sense-bearing elements extracted from the real text. Using them as guidance ensures semantic integrity and relevance of the generated text. We attempt to control the proximity of generated data to original data and vary the quantity of phrases provided. We seek to compensate missing information with the clinical information related to patients and their hospital admissions (Fig. 1).

We perform an extensive intrinsic evaluation of generated text to (1) measure text preservation by using a range of shallow automatic metrics, (2) measure how much the models memorise information from the training data, and (3) assess the clinical validity of the generated text through human evaluation.

**Fig. 2:**
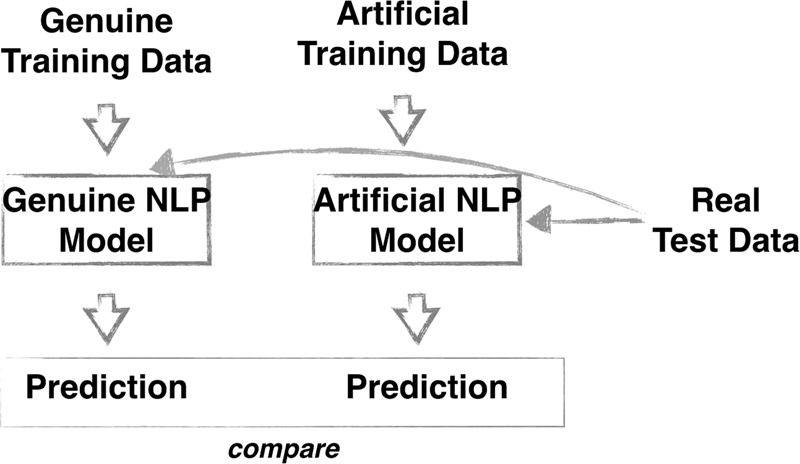
Overview of the extrinsic evaluation procedure. An NLP model is built on 1) genuine data and 2) artificial data. Both models are tested on real (genuine) test data. Comparing these results gives an indication of the usefulness of using artificial data for NLP model development.

At the extrinsic evaluation step, we use generated data in a text classification experiment, involving several standard NLP models (both neural and non-neural). Using the original test data, we assess performance of each model trained using artificial data against the one built using genuine data (Fig. 2). Useful artificial data models should demonstrate similar performance results to models developed on genuine data. Most importantly, the artificial models should correctly show performance differences between different classification algorithms so that in a real-life scenario, NLP methods developed by external providers where models have been developed on artificial data would perform similarly on the genuine data.

Our study is an important first step towards our long-term goal of generating artificial data that: (a) are statistically close to original data and hence useful for NLP development, and (b) can be released to the wider research community under appropriate, but less strict governance regulations as they should not retain rare or unusual information from the original data that could pose any disclosure risks.

Our main goals are: (1) test the hypothesis that statistically and clinically valid data could be generated with our proposed approach; (2) test the hypothesis that the generated medical data could be useful for downstream NLP tasks; (3) test whether this generation process could be efficiently controlled by key phrases with the potential to control the risk of rare information seeping through into the generated artificial data.

It should be emphasised that data we use in this study is already de-identified (defined as removing protected health information (PHI)). Thus, in our study, the focus is not on de-identification per se, rather, it is to try to quantify and assess whether other unusual or rare information from the already de-identified input data leaks into the synthetic data, and with that analyse and reason about the potential impact of this for releasing this type of data to the research community with less strict governance procedures.

## Results

### Datasets

We use EHRs from two different databases: a MHR database and the MIMIC-III database^7,8^. Two text classification tasks are studied: diagnosis code and phenotype, further described below.

#### CRIS MHR dataset

We use discharge summaries of pseudo-anonymised and de-identified MHRs from the Clinical Record Interactive Search (CRIS) database at the South London and Maudsley (SLaM) NHS Trust^9^. The de-identification was performed with respect to patient identifiers (e.g., patient, and relative names and phone numbers, etc.) that were replaced with placeholders^10^. From all the discharge summaries present in the database, we retain only those coded with common mental health ICD-10 diagnoses.

For the text generation experiments, the dataset is divided into training, validation, and test sets (*train-gen-mhr*, *val-gen-mhr*, and *test-gen-mhr*, respectively). We report the frequency of ICD-10 codes in the test set (Table 1). The final training set (*train-gen-mhr*) consists of 24,273 patient IDs, 537K lines, and 12M tokens; the validation set (*val-gen-mhr*) consists of 1348 patient IDs, 30K lines, and 653K tokens; and the test set (*test-gen-mhr*) consists of 1349 patient IDs, 29K lines, and 659K tokens.

**Table 1:** Mental health diagnoses from discharge summaries in the CRIS database. We report frequency for *test-gen-mhr*.

ICD-10	Description	Freq (%)
F20	Schizophrenia	29
F32	Major depressive disorder, single episode	21
F60	Specific personality disorders	16
F31	Bipolar affective disorder	14
F25	Schizoaffective disorders	11
F10	Mental and behavioural disorders due to use of alcohol	9

Ten percentage and 20% of *test-gen-mhr* are randomly selected for the development and test purposes, respectively, for the text classification task (diagnosis code). This results in *train-class-mhr*, *dev-class-mhr*, and *test-class-mhr*.

#### MIMIC-III dataset

We use the MIMIC-III dataset for a phenotyping classification task described by Gehrmann et al.^11^. Phenotyping is the task of determining whether a patient has a medical condition or is at risk for developing one. This dataset includes discharge summaries of ~1000 patients annotated with 13 phenotypes (e.g., advanced cancer, chronic pain, obesity, depression, etc.). For our generation experiments, we extract all the MIMIC-III discharge summaries of patients with the three first diagnoses (represented by the two first characters of each respective ICD-9 code), matching at least one sequence of the three first diagnoses for the patients from our phenotyping dataset. Thus, our text generation dataset does not contain the patients from the phenotyping dataset. The de-identification of the MIMIC data was performed with respect to PHI (e.g., to doctor names and years of dates in addition to patient information) that were replaced with placeholders^12^.

All the extracted data is then split into two subsets: *train-gen-mimic* (9767 patient IDs, 10,926 admission IDs, 1.2M lines, and 20M tokens) and *val-gen-mimic* (126 patient IDs, 132 admission IDs, 13K lines, and 224M tokens). The annotated phenotyping dataset (*test-gen-mimic*, 1045 patient IDs, 1560 admission IDs, 183K lines, and 3M tokens) is used as test set. The phenotyping dataset was initially collected for MIMIC-II. We could not hence reliably identify text fields in MIMIC-III for records with duplicated admission IDs. We simply merged those records together giving preferences to annotations with a higher rate of positive labels. This resulted in a small reduction of the initial dataset (<1%). For the phenotype classification task, 10% and 20% of *test-gen-mimic* are randomly selected for the development and test sets, respectively. This results in the three following sets: *train-class-mimic*, *dev-class-mimic*, and *test-class-mimic*.

### Intrinsic evaluation

In our experiments, we attempt to control the proximity of the generated data to the original data, and vary the quantity of phrases provided. We seek to compensate missing information with the clinical information related to patients and their hospital admissions: patient gender and age, Boolean switch indicating death, diagnosis description, timestamp of a record relative to admission date, record section (summary, discharge plan, or comments), and the ordinal number of a sentence in a section.

Overall, we test the following three experimental setups: (a) artificial text generation using all the extracted key phrases (*all*), (b) using a set of best-scored key phrases plus clinical information (*top+meta*), and (c) using the one best-scored key phrase per sentence plus the clinical information (*one+meta*). As a baseline method, we take all of the extracted key phrases (*key*, reproducing the inputs instead of generating outputs). This baseline represents the worst possible generation model that copies the input without generating any context to it.

The intrinsic evaluation step allows to determine some types of differences and similarities between the generated text and the authentic text (that is used for the extraction of key phrases that serve as input to the model). We report perplexity (PPL); a set of metrics comparing lexical content of original and generated data: ROUGE-L, BLEU, and TER; as well average sentence lengths. PPL reflects the confidence of the model in the produced output (the higher the value the lower the confidence). ROUGE-L^13^ measures the longest in-sequence common *n*-gram recall between generated and original text, BLEU^14^ — *n*-gram precision, whereas TER^15^ — the minimum number of edits (substitution, insertion, deletion, and shift of a word) required to change a generated sentence so that it exactly matches a genuine one. Overall, ROUGE-L measuring recall and BLEU measuring precision are complementary, whereas TER gives an idea of the amount of changes performed to the real text.

Table 2 reports the results of the intrinsic evaluation for both generation test sets. As expected, the more information we input, the closer the generated text is to the original one: *all* provides the closest results (av. ROUGE-L = 0.8 for both test sets). *key* sentences are the shortest, which means that the risk of these generated sentences losing important information is higher.

**Table 2:** Qualitative evaluation and average sentence lengths on the CRIS data (*test-gen-mhr*) and the MIMIC-III data (*test-gen-mimic*). Models providing data closest to the original data according to all the scores are highlighted in bold.

	PPL	ROUGE-L↑	BLEU↑	TER↓	∼*l*
*test-gen-mhr*					
genuine	−	−	−	−	22.44
* all*	7.24	0.76	40.88	0.39	17.84
*** top+meta***	**15.57**	**0.58**	**25.10**	**0.59**	**15.02**
* one+meta*	37.46	0.40	10.29	0.80	10.63
* key*	−	0.58	7.75	0.56	10.21
*test-gen-mimic*					
genuine	−	−	−	−	17.55
* all*	3.22	0.81	53.45	0.31	14.5
*** top+meta***	**5.14**	**0.68**	**37.28**	**0.49**	**12.39**
* one+meta*	9.75	0.47	16.66	0.74	9.72
* key*	−	0.59	8.70	0.56	7.94

The most promising results are obtained from the *top+meta* model. This model results in longer sentences compared to the other models, while still retaining the most balanced scores across the other metrics.

**Fig. 3:**
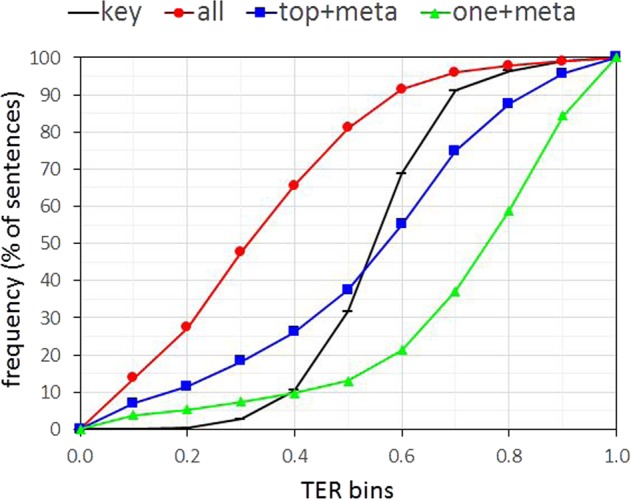
Cumulative distributions (CDFs) of the TER bins for the *key*, *all*, *top+meta*, and *one+meta* sentences for *test-gen-mhr*. *X*-axis plots TER bins. *Y*-axis—respective cumulative frequencies of the *test-gen-mhr* sentences.

Figure 3 reports the cumulative distributions (CDFs) of the TER bins for the *key*, *all*, *top+meta*, and *one+meta* sentences for *test-gen-mhr*. *all* practically restores the majority of the original sentences (only 20% of the *test-gen-mhr* sentences have high TER ≥ 0.5). Input with minimum original words on the other hand (*one+meta*), results in 85% of the generated sentences having high TER ≥ 0.5. Finally, the distribution of the *top+meta* TER scores is almost uniform. A similar observation is made for the *test-gen-mimic* results.

As we are interested in keeping only the key meaning of the original text and modifying its context, in our further analysis, we focus on the text generated by *top+meta* and *one+meta*.

### Memorisation evaluation

According to the standard procedures our generation models are trained to recreate real data, there is thus an actual risk that the models will overfit, memorise training data, and produce disclosing patient information in their outputs. In the design of our experiments, we have left the identification and masking of PHI cases (e.g., names and addresses) to be handled in the preprocessing. The data we work with were already de-identified using bespoke, standard procedures. In addition, the least frequent words were removed to not be included in the model vocabulary. However, also indirect references to some rare events, e.g., “the accident was widely reported in the press” in the generated text could potentially identify a patient. As mentioned, in our study, the focus is on trying to assess the risk of such unusual or rare information from the already de-identified input data leaking into the synthetic data. For the best-performing *top+meta* *train-gen-mhr* model, we assess how well our model memorises the training data.

Inspired by Carlini et al.^16^, we regenerate sentences from the training data that contain rare (lower frequency quartile) *n*-grams. We experiment with 2-grams and 3-grams as the average length of extracted key phrases, as well as with 5-gram (as longer text spans). For each *n*, we randomly select 1K unique sentences with a rare *n*-gram each. In a contrastive setting, we also randomly select 1K unique sentences with a high-frequency *n*-gram each (upper frequency quartile). Note that 1-grams with frequency 1 were already excluded from the training data to limit the vocabulary size.

We first analyse how many of those selected *n*-grams are already extracted as key phrases in the input (%, in). Table 3 shows that low-frequency *n*-grams are extracted as key phrases more often than high-frequency *n*-grams: e.g., 16% of all the high-frequency 2-grams and 40% of all the low-frequency 2-grams. In the generated output (%, out), both low-frequency and high-frequency 2-grams are equally present (48% for both cases). This means that only 8% of the low-frequency 2-grams are memorised and restored in the output, while 32% of the high-frequency 2-grams is restored in the output.

**Table 3:** Memorisation assessment for 1K samples per *n*-gram group in the *top+meta* *train-gen-mhr* model. *high* denotes *n*-grams from the upper frequency quartile; *low*—*n*-grams from the lower frequency quartile; *%,in* denotes percentage of target *n*-grams in the input key phrases; and *%,out*—in the respective generated output. Highest PPL values are highlighted in bold.

		2-gram		3-gram		5-gram	
		High	Low	High	Low	High	Low
%, In		16	40	4	12	0.3	0.8
%, Out		48	48	43	34	41	29
PPL, K		18	**25**	17	**24**	21	**24**

Presence/absence of an *n*-gram in the output is however dependent on the decoding procedure. Thus, we also report the average PPL values that reflect the confidence of the model in reproducing samples from the training set (the higher the value the lower the confidence). Table 3 shows that the PPL values tend to show higher values for restoring sentences with low-frequency *n*-grams, demonstrating the challenge of the task.

Overall, we consider there is a low risk that our model reveals identifiable information. The model is not prone to overfitting. The main proportion of rare information is provided with the input key phrases and can be controlled. We imagine applying filters (e.g., pretrained classifiers) to model inputs that would detect rare key phrases, containing information on rare diseases, religion, race, or sexuality, etc. Also not many *n*-grams could be potential identifiers. For instance, ~20% of tokens in rare 2-grams restored in the output are stopwords, punctuation or numerical values that could be filtered out even with a rule-based procedure.

### Human evaluation

For the human evaluation task, we made an assumption that good artificial text should either: (1) keep the main clinical meaning of the genuine text or (2) modify it so that it remains valid (given the associated diagnosis). Hence, we defined seven fine-grained annotation categories that reflect the proportion of the original meaning preserved (Table 4): from meaning fully preserved up to meaning modified, contradicts the diagnosis, and makes no sense from the clinical point of view. These are further grouped into four more generic categories: SAME, GOOD, BAD/IRRELEVANT, and NO SENSE.

**Table 4:** Annotation categories for the human evaluation of the meaning of the generated text.

	Category	Group
1	Fully preserved	SAME
2	Preserved, details omitted	GOOD
3	Modified, does not contradict the diagnosis	GOOD
4	Modified, contradicts the diagnosis	BAD/IRR
5	Modified, irrelevant	BAD/IRR
6	No clinical sense	NO SENSE
7	Incomprehensible	NO SENSE

Annotations were carried out by Joyce Kam (annotator 1), Somain Verma (annotator 2), and Lucia Yin (annotator 3), all medical students, native English speakers, experienced in annotating clinical text, for *test-gen-mhr* for both *top+meta* and *one+meta*. The students were provided with a file per discharge summary containing parallel genuine and generated text, as well as the diagnosis information.

**Fig. 4:**
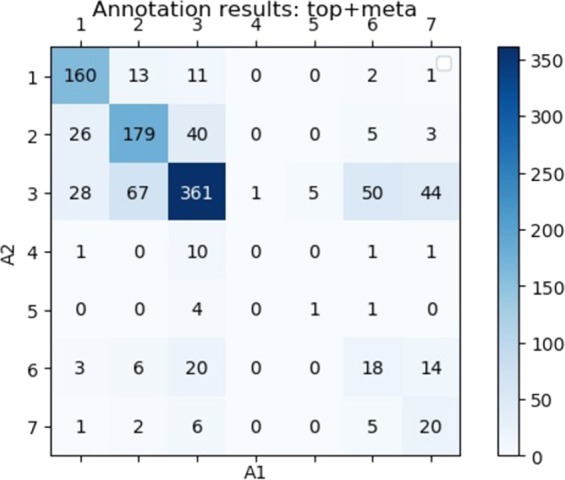
Matrix of inter-rater annotation agreement for 1K *top+meta* sentences. For each document, we defined A1 as the first annotator and A2 as the second annotator. Each cell in the matrix represents the number of sentences marked by an annotator with a certain category (as defined in Table 4).

A total of 120 documents were double-annotated for both *top+meta* and *one+meta* (~1K sentences per setup). Figure 4 shows the annotation results for *top+meta*. For each document, we defined A1 as the first annotator and A2 as the second annotator. Annotations were carried out as follows: annotator 1 vs. annotator 2, 38% of the data; annotator 1 vs. annotator 3, 51% of the data; and annotator 2 vs annotator 3, 11% of the data. We measure the inter-annotator agreement using accuracy and Cohen’s kappa coefficient (*κ*) over groups. The accuracy is 0.78, for *top+meta*, and 0.87, for *one+meta*; *κ* is 0.54 for *top+meta*, and 0.49 for *one+meta*. Results with both scores indicate a sufficient agreement between the annotators. Computing agreement per annotator pair did not change results significantly. For both *top+meta* and *one+meta*, the most frequent categories are “Modified, does not contradict the diagnosis” (3) (49% and 66%, respectively) and “Preserved, details omitted” (2) (24% and 24%, respectively). Most of the disagreement is between the groups GOOD and NO SENSE (*κ* = 0.37, fair). Annotations for category 4 (contradiction to the diagnosis) are very rare (~1% for both *top+meta* and *one+meta*) and were mainly assigned by only one annotator.

As reported by the annotators, the quality of the generated text was in general high as compared to their expectations. However, the annotation task was considered challenging for some specific cases (e.g., long sentences that were partially incomprehensible, but could still make sense).

In Table 5, we report seven examples of generated sentences (all paraphrased), with corresponding annotation categories. The second and third examples represent “good cases”, as generated texts are clinically valid and do not contradict the diagnosis. In the remaining sentences, instead, we have a few undesirable cases: the fourth sentence contradicts the diagnosis, while the sixth sentence does not make sense from the clinical point of view. Finally, the last sentence is not comprehensible at all. In Table 6, we report two examples of generated sentences (all paraphrased), with disagreement on annotation categories. In the first case, the artificial sentence includes a nonsensical fact; however, the main symptom is retained (“NO SENSE” vs. “GOOD”). In the second case, it seems as though one symptom is introduced in the artificial sentence; however, the wording is not too far from the original one (“BAD/IRR” vs. “GOOD”).

**Table 5:** Examples of artificial sentences and respective real sentences (all paraphrased) for *test-class-mhr*.

Fully preserved	real	There was no clear evidence that he was responding to unseen stimuli.
	art.	No clear evidence of responding to unseen stimuli.
Preserved, details omitted	real	He did not have a clear understanding of why he was there or what was the problem with him.
	art.	He has no clear understanding why he is there.
Modified, does not contradict the diagnosis	real	That afternoon police were called after she assaulted her mother.
	art.	This afternoon police were called by her mother.
Modified, contradicts the diagnosis	real	He was not experiencing low mood or anhedonia and therefore does not meet the criteria for depressive disorder.
	art.	Today he continues to experience low mood and anxiety.
Modified, irrelevant	real	Her partner wants him to stay with him.
	art.	Her partner wants him to get out of bed.
No clinical sense	real	She acknowledged that paracetamol overdose could damage her liver.
	art.	Paracetamol overdose could damage her shoulder.
Incomprehensible	real	This relapse of heavy drinking may have been caused by the disruption of her accommodation relocation.
	art.	It was felt that heavy drinking may not be a accommodation relocation.

**Table 6:** Examples of disagreements on artificial sentences and respective real sentences (all paraphrased) for *test-class-mhr*.

GOOD vs. NO SENSE	real	When he was approached by the police, he started removing his trousers and becoming quite aggressive.
	art.	He had started removing the hair of the window, becoming quite aggressive.
GOOD vs. BAD/IRR	real	She appeared as though she felt under threat but the ward was very chaotic at that time with loud bangs.
	art.	Chaotic and loud bangs in her interactions.

### Extrinsic evaluation

Table 7 shows our text classification results (*F*1-scores) for *test-class-mhr*. Globally, we compare performances of the three classification models: (a) a Latent Dirichlet allocation (LDA) model with 150 topics^17^ trained on *train-gen-mhr*/ *train-gen-mimic* with the Random Forests (RF)^18^ algorithm; (b) an *n*-gram (up to 5) bag-of-words (BoW) model with RF; and (c) a Convolutional Neural Network (CNN) inspired by Kim^19^. Within each model, we compare each distribution of *F*1-scores produced using a synthetic training set to a distribution for the original data, and report the two-sample Kolmogorov–Smirnov (2S-KS) equality test for not normally distributed independent samples. *F*1-scores do not compute means, the common practice is to assume that they are not normally distributed^20^. We perform two types of comparisons using 2S-KS: (a) comparisons between different models trained with the same type of data (e.g.,: LDA genuine vs. BoW genuine); and (b) comparisons within a model trained with the different types of data (e.g., LDA genuine vs. LDA *top+meta*, LDA genuine vs. LDA *one+meta*, etc.). For example, Table 7 reports the 0.51 *p*-value for LDA *top+meta*, which is computed against the LDA genuine sample. The same table reports the significance in performance differences (marked with *) for LDA genuine vs. BoW genuine, and LDA *top+meta* vs. BoW *top+meta*, etc.

**Table 7:** Text classification results (*F*1-scores) for *test-class-mhr* (fivefold CV; results averaged per class). We use 2S-KS test for (a) comparisons between models trained with the same type of data; * marks statistically significant improvements for LDA over BoW, and CNN over LDA (*α* = 0.05, *n*_1_ = *n*_2_ = 30); (b) comparisons within a model trained with the different types of data (column KS test). Models using less than *all* key phrases that provided results closest to those with real data are highlighted in bold. We also report results of our ablation experiments when the training data contain only the context of key phrases, real, or generated.

	ICD-10							
	*F*20	*F*32	*F*60	*F*31	*F*25	*F*10	**av**.	KS test, (*D*, *p*-value)
BoW								
genuine	0.47	0.31	0.32	0.20	0.14	0.24	**0.28**	
* all*	0.47	0.33	0.27	0.23	0.17	0.23	**0.28**	0.07, 0.88
* top+meta*	0.48	0.36	0.29	0.20	0.14	0.26	**0.29**	0.09, 0.61
*** one+meta***	**0.46**	**0.34**	**0.29**	**0.23**	**0.14**	**0.26**	**0.29**	**0.07**, **0.80**
* key*	0.47	0.27	0.26	0.11	0.12	0.23	**0.24**	0.17, 0.02
LDA								
genuine*	0.55	0.47	0.35	0.32	0.25	0.40	**0.39**	
all*	0.55	0.44	0.35	0.31	0.26	0.37	**0.38**	0.11, 0.35
top+meta*	**0.52**	**0.43**	**0.37**	**0.29**	**0.25**	**0.40**	**0.38**	**0.09**, **0.51**
one+meta*	0.50	0.45	0.36	0.28	0.23	0.39	**0.37**	0.14, 0.10
key*	0.54	0.45	0.38	0.30	0.24	0.40	**0.39**	0.07, 0.88
CNN								
genuine*	0.66	0.59	0.51	0.37	0.23	0.53	**0.48**	
all*	0.65	0.57	0.47	0.27	0.24	0.50	**0.45**	0.14, 0.10
top+meta*	**0.63**	**0.55**	**0.45**	**0.31**	**0.23**	**0.42**	**0.43**	**0.20, 4e−3**
one+meta*	0.59	0.52	0.42	0.25	0.15	0.43	**0.39**	0.22, 1e−3
* key*	0.57	0.34	0.33	0.23	0.20	0.35	**0.34**	0.37, 1.9e−09
No key phrases								
CNN								
genuine	0.48	0.34	0.22	0.22	0.15	0.12	0.25	
* top+meta*	0.30	0.30	0.09	0.25	0.09	0.03	0.18	0.24, 2.7e−04
LDA								
genuine*	0.41	0.40	0.32	0.22	0.20	0.26	0.30	
top+meta*	0.29	0.37	0.28	0.23	0.14	0.25	0.26	0.23, 4.4e−04

CNN is the best-performing model, showing a significant improvement over LDA, which in turn significantly outperforms BoW (CNN *F*1-score_av_ = 0.48, LDA F1-score_av_ = 0.39, and BoW *F*1-score_av_ = 0.28, all genuine). Artificial data from our *top+meta* and *one+meta* methods are useful for our chosen downstream NLP tasks, and manage to maintain model performance differences. Similar tendencies are observed for all the three models in spite of their intrinsic differences: BoW is focused on *n*-gram counts, LDA is topic oriented with the focus on keywords and CNN combines the adjacent distributed representations of words to analyse concepts. Not surprisingly, the *all* setup provides the results closest to the original.

On the other hand, the *key* baseline (only all the key phrases without text generation) performs poorly for two models out of three: LDA and CNN. For CNN, it even distorts the results: *key* LDA outperforms *key* CNN (*ΔF*1-score_av_ = 0.05), whereas CNN outperforms BoW for the real data. Thus, in most cases our generation methods manage to capture useful information for downstream NLP tasks. *top+meta*, where only ∼31% of original words per sentence is used, performs consistently well for two out of three models (LDA and CNN).

We also analyse errors of the best-performing genuine and *top+meta* CNNs. Firstly, we focus on the “bad errors” of both models: false negatives (FNs) and false positives (FPs), where the model has high confidence in the wrong result. The majority of “bad errors” are due to FPs (420 and 256, for *top+meta* and genuine, respectively) rather than FNs (110 and 127, for *top+meta* and genuine, respectively). Moreover, 42–45% of the genuine errors are found in *top+meta* as well. As an interesting result, while the number of FPs was higher for *top+meta*, the genuine model resulted in a slightly higher number of FNs.

In terms of precision and recall, the genuine model had a slightly higher recall than precision, while the *top+meta* model showed comparable values. Overall, the *top+meta* CNN reflects the behaviour of the genuine CNN, also when looking at the different diagnoses, and even potentially improves it by slightly reducing its FN count.

As a sanity check, we perform a series of ablation experiments to verify if the real key phrases in the artificial data influence the classifiers. Again for the *top+meta* setup, we remove the common key phrases from both the genuine and artificial data, and compare the performance of our classifiers. We focus on the best-performing LDA and CNN. Table 7, last four lines, shows the results of those experiments. We again observe comparable performances for the genuine and artificial models. This confirms that our artificial data captures relevant information.

Finally, Table 8 shows our text classification results for *test-gen-mimic*. CNN is again the best-performing model showing a significant improvement over BoW, which significantly outperforms LDA (CNN *F*1-score_av_ = 0.46, BoW *F*1-score_av_ = 0.34, and LDA *F*1-score_av_ = 0.23, all genuine). Artificial data from *top+meta* and *one+meta* again manage to correctly reveal performance differences between models. *top+meta* has the optimal performance for BoW and CNN. Both *top+meta* and genuine samples have relatively high probabilities to belong to the same distribution with *p*-values of 0.28 and 0.13 for BoW and CNN, respectively.

**Table 8:** Text classification results (averaged *F*1-scores) for *test-class-mimic*. We use 2S-KS test for (a) comparisons between different models trained with the same type of data. * Marks statistically significant improvements for LDA over BoW, and for CNN over LDA (*α* = 0.05, *n*_1_ = *n*_2_ =65); and (b) comparisons within a model trained with the different types of data (column KS test). Models using less than *all* key phrases that provided results closest to those with real data are highlighted in bold.

	**av**.	KS test, (*D*, *p*-value)
LDA		
genuine	0.23	
* all*	0.21	0.22, 0.08
*** top+meta***	**0.21**	**0.21, 0.08**
* one+meta*	0.21	0.23, 0.05
* key*	0.13	0.54, 5.15e−09
BoW		
genuine*	0.34	
all*	0.32	0.14, 0.53
top+meta*	**0.31**	**0.17, 0.28**
one+meta*	0.27	0.29, 0.01
key*	0.30	0.19, 0.20
CNN		
genuine*	0.46	
all*	0.45	0.12, 0.68
top+meta*	**0.40**	**0.20, 0.13**
one+meta*	0.36	0.35, 4e−4
* key*	0.24	0.59, 1.5e−10

## Discussion

We present an approach to generate clinical documents (EHR discharge summaries), based on the Transformer model. To maintain semantic coherence at a paragraph level, the sentence by sentence generation is guided by key phrases. Different configurations of the amount of key phrases are applied, as well as clinical information, to investigate how much of the original data is needed to generate useful artificial data. We demonstrate the validity of our approach on two EHR datasets: on discharge summaries from a large MHR system, and discharge summaries from an intensive care unit. MHR notes are particularly challenging as they contain more complex narratives, and this type of clinical documentation tends to rely less on structured coding.

An extensive intrinsic evaluation shows that the *top+meta* model, which uses very little information from the original text, memorises few rare *n*-grams. This is promising in terms of assessing the risk of these models retaining information from the original data that should ideally be rephrased, to ensure that the artificial data minimises any traces of the original data. The clinical validity is at the same time to a large extent preserved, as indicated by the human evaluation task.

Furthermore, an extrinsic evaluation is performed in downstream NLP text classification tasks with two datasets: diagnosis code and phenotype classification. Using the artificial data as training data leads to comparable results as to those obtained from using the original data.

We have created a light-weight solution that any holder of clinical data could apply, in order to generate synthetic data to outsource NLP algorithm development. Clinical institutions do not often have the internal expertise for NLP development and getting the appropriate authorisation to allow this data to be accessed by external organisations is often time-consuming. That is why we show that comparison of NLP models trained with synthetic data holds for real data. The main purpose is to speed up the external NLP development process with some kind of proxy of real data and get a fair model faster, while still adhering to governance procedures in using clinical data. Of course, in a clinical production setting, these best NLP models should be rebuilt with real data and properly tested.

We demonstrate that our methodology is not prone to overfitting and the data it generates can easily be shaped by the input selection. This means that the sensitive information in the original training data can be efficiently protected. Our findings have important implications for our long-term goal to generate artificial data that can be released to the wider research community.

There are several directions to take our work further. The methodology of generation with key phrases could be replaced by other types of modelling that minimises genuine input, e.g., adversarial learning approaches. Advances in privacy preserving algorithms, such as those by Sánchez and Batet^21^ or Anandan et al.^22^ could inspire alternative approaches to assess the amount of the remaining potentially identifiable information.

Finally, we have investigated only one downstream NLP task. Looking forward, a more universal approach to generate data for other clinical NLP tasks (e.g., information extraction or temporal modelling) is needed. For such other NLP tasks, this text generation approach might not be optimal, as other constraints are imposed. Moreover, other types of clinical use cases might require multiple documents per patient; how to address longitudinal coherence would need further analysis. Assessing clinical validity for other tasks might also require defining the human evaluation task slightly differently.

Having ways of generating artificial clinical data from already PHI de-identified original data that further alleviates the risk of containing any sensitive information could have a huge impact on the development of novel NLP, and other data science approaches for analysing EHR data, particularly by making data more widely available to the research community. This, in turn, could have significant impact in using retrospective, secondary healthcare data for translational research that can be used to improve quality of care for patients. To date, there are no agreed-upon metrics and thresholds to use for assessing the risk of revealing identifiable information from free-text data, but more importantly, there are also very few studies that provide an evaluation of how realistic artificially generated data are, and the impact of this for downstream tasks. Our study is a first step in addressing these issues, and we will further evaluate and analyse these questions initially by organising a workshop with service users, clinical and computer science researchers, as well as information governance practitioners in healthcare services.

## Methods

### Ethics approval information

The de-identified CRIS database has received ethical approval for secondary analysis: Oxford REC C, reference 18/SC/0372. The data are used in an entirely anonymised and data-secure format and therefore, under UK law, does not require informed consent from patients whose data are represented here. Instead, patients are routinely informed of the data resource and have the opportunity to opt out (taken up by four people to date). CRIS data is made available to approved researchers working on approved projects. Projects are approved by the CRIS Oversight Committee, a body setup by and reporting to the SLaM Caldicott Guardian. Researchers are approved by application to SLaM NHS Trust. The study protocol presented here is CRIS approved project reference number 18-103 (“Towards Shareable Data in Clinical Natural Language Processing: Generating Synthetic Electronic Health Records”). No further approvals were required for work on this nature.

The study has been carried out in accordance with relevant guidelines and regulations for the MIMIC-III data.

### Text generation models

In our attempt to find an optimal way to generate artificial EHRs, we experiment with the neural Transformer model^23^, an ED architecture^24,25^, state-of-the-art in text generation. In this architecture, the decoder is a conditional Language Model. It generates a new word at each timestep taking into account the previously generated words, as well as the information provided by the encoder (a sequence of hidden states — roughly speaking, a set of automatically learned features). For different tasks, the input to the encoder may vary: questions for question–answering, source text for MT, story prompts for storytelling, etc. In this work, we follow the approach of Peng et al.^26^ and guide the generation of EHRs with the help of key phrases. These key phrases are sense-bearing elements: using them as guidance ensures semantic integrity and relevance of the generated text.

We extract key phrases at the paragraph level, match them at the sentence level and further use them as inputs into our generation model. Thus, each paragraph is generated sentence by sentence (standard practice in text generation) but taking the information ensuring its integrity into account. In short, the model fills related textual context around given key phrases.

Key phrases are extracted from each original paragraph of *train-gen-mhr* or *train-gen-mimic*. We use the *Rake* algorithm^27^. For both datasets, this results in approximately five key phrases with an average length of two words per sentence (*all* setup). The top-scored key phrases make around three key phrases per sentence (*top+meta* setup). One best key phrase per sentence is chosen for *one+meta*. The key phrases and the clinical information are simply concatenated in the input to the model (e.g., input “*F*20 *F* 23 female allergies”, see Fig. 1). We do not mark borders of key phrases. The clinical information is represented with mostly abbreviations each having a separate embedding.

We train our models for the gap-filling task. In the input, we have the clinical information and the key phrases and in the output, we have the full original EHR record. For example, a training example: input “*F*20 *F* 23 female allergies” -> “A female in her twenties has allergies” (Fig. 1). The model is trained to restore the text highlighted in bold.

### Text classification models

For both the CRIS and MIMIC classification datasets, we cast detection of each diagnosis/phenotype as a binary classification task to better analyse each result in isolation. For small and unbalanced clinical training data, such as the CRIS data in this study, a best practise is to use *k*-fold cross validation (CV, where *k* is usually 5 or 10) to ensure that every example appears both in the training and test data. In this way, any important information will not be missed from the training data of a model, whereas with bigger data there is more chance to have a proper distribution of information for both training and test data. For the MIMIC phenotyping task, the test set is released together with the data. We follow Gehrmann et al.^11^, reuse the provided test set and cast the task as a binary classification task. Considering the random initialisation of parameters in our models, for each experiment we retrain the model five times to increase reliability of our estimations. The three classification models (BoW, LDA, and CNN) are chosen as three state-of-the-art models at different stages of the development of NLP, in the order of the typical performance improvement. For the KS test computations, we consider the data points from each five runs of each model for binary prediction training as a single sample.

### Reporting summary

Further information on research design is available in the Nature Research Reporting Summary linked to this article.

## Supplementary information

reporting summary

## Data Availability

On request, and after appropriate arrangements, the CRIS data and modelling employed in this study can be viewed within the secure system firewall (details in section Ethics Approval Information). Access to the MIMIC-III data was obtained following the Physionet requirements: https://mimic.physionet.org/gettingstarted/access (accessed 18 October 2019.) The study has been carried out in accordance with relevant guidelines and regulations.

## Code availability

Our models are trained on real, de-identified, but protected data (CRIS data are protected under a governance model, MIMIC data requires authorisation). Thus our trained models can not be made publicly available online. There are multiple implementations of the Transformer model publicly available, thus the procedure to create a model is straightforward.

We train Transformer models as provided by the OpenNMT-py toolkit (version 0.2.1)^28^ with default parameters. Each model is trained for 30K steps. We noticed that this quantity of epochs is necessary for the stabilisation of the model PPL. For all the generation models we use a vocabulary of around 50K most frequent words. Outputs are produced with the standard beam decoding procedure (default beam size 5). We use the implementation of the *Rake* algorithm available at https://github.com/csurfer/rake-nltk.

Data extraction and preprocessing scripts are publicly available online: https://github.com/KCL-Health-NLP/artificialMHR. Our preprocessing pipeline, including sentence detection uses the spaCy toolkit (https://spacy.io/, version 2.0.18). We lowercase all the text. In addition, we replace dates with a placeholder *date*. We discard all the sentences with length under five words. We replace all the out-of-vocabulary words and words with frequency 1 with the *UNK* placeholder.

To build our text classification models, we use the implementation of LDA available in the Gensim toolkit (version 3.6.0)^29^ with 150 topics. All other parameters are those provided by default. The model is trained on *train-gen-mhr* and *train-gen-mimic* respectively with stopwords removed as defined by the respective NLTK list (version 3.4)^30^.

For both LDA and BoW models, we use the RF implementation available in *Scikit-learn* (version 0.20.2)^31^ with the following parameters: the balanced subsample class weight and a maximum depth of 2. All other parameters are those provided by default.

For the CNN model, the word embedding dimensionality is 500. Both convolution layers have ten hidden units and filters of three and eight. The size of max-pooling layers is set to two. We use two dropout layers (one before and one after the convolution layers) with probabilities of 0.5 and 0.8. The last dropout layer is followed by a dense layer. The size of hidden units of the dense layer is 50. We use random uniform initialisation and L2 regularisation. To optimise the usage of computational resources, in each experiment we fix the size of a record to the upper quartile of the distribution of record length values (in words). All the hyperparameters are tuned on the validation sets (*dev-class-mhr* and *dev-class-mimic* respectively). The model uses Word2Vec embeddings^32^ pretrained using the Gensim toolkit (version 3.6.0) on *train-gen-mhr* and *train-gen-mimic* respectively. The CNN model is implemented using the Keras toolkit (https://keras.io, version 2.2.4).

Experiments with the CRIS data are performed on an Azure Tesla GK210 GPU (NC6 configuration with the Tesla K80 Accelerator), experiments with the MIMIC data – on a GeForce GTX 1070 8Gb GPU.
